# Effects of Alloying Elements (C, Mo) on Hydrogen Assisted Cracking Behaviors of A516-65 Steels in Sour Environments

**DOI:** 10.3390/ma13184188

**Published:** 2020-09-21

**Authors:** Jin Sung Park, Jin Woo Lee, Joong Ki Hwang, Sung Jin Kim

**Affiliations:** 1 Department of Advanced Materials Engineering, Sunchon National University, Jungang-ro, Suncheon, Jeonnam 57922, Korea; pjs1352@naver.com; 2 POSCO Steel Solution Center, Pohang, Gyungbuk 790-704, Korea; jwlee36@posco.co.kr; 3 Department of Mechanical Engineering, Tongmyong University, Busan 48520, Korea; jkhwang@tu.ac.kr

**Keywords:** steel, hydrogen induced cracking, sulfide stress cracking, sour, hydrogen permeation, diffusion, corrosion

## Abstract

This study examined the effects of alloying elements (C, Mo) on hydrogen-induced cracking (HIC) and sulfide stress cracking (SSC) behaviors of A516-65 grade pressure vessel steel in sour environments. A range of experimental and analytical methods of HIC, SSC, electrochemical permeation, and immersion experiments were used. The steel with a higher C content had a larger fraction of banded pearlite, which acted as a reversible trap for hydrogen, and slower diffusion kinetics of hydrogen was obtained. In addition, a higher hardness in the mid-thickness regions of the steel, due to center segregation, resulted in easier HIC propagation. On the other hand, the steel with a higher Mo content showed more dispersed banded pearlite and a larger amount of irreversibly trapped hydrogen. Nevertheless, the addition of Mo to the steel can deteriorate the surface properties through localized pitting and the local detachment of corrosion products with uneven interfaces, increasing the vulnerability to SSC. The mechanistic reasons for the results are discussed, and a desirable alloy design for ensuring an enhanced resistance to hydrogen assisted cracking (HAC) is proposed.

## 1. Introduction

Hydrogen assisted cracking (HAC), which is classified into hydrogen induced cracking (HIC) and sulfide stress cracking (SSC) by the presence and absence of applied stress, is one of the most important issues in the petrochemical industries [1,2,3,4,5]. In a recent refining system for low-grade crude oil, the refining temperature and pressure required have increased remarkably. The sensitivity of material deterioration, caused by the inflow of hydrogen atoms in the steel structures, is becoming higher than in the past [6,7]. Accordingly, considerable efforts have been made to develop the steel used as pressure vessel facilities with superior resistance to HAC [7,8,9,10]. For this purpose, a variety of technical solutions, such as de-phospho/sulfu-rization of molten steel [11,12], Ca-treatment [13], cast steel soft reduction [14], and thermal mechanical controlled process (TMCP) [15,16,17], have been proposed. Microstructural modifications by reducing the number of second phases, such as retained austenite (RA), martensite-austenite constituents (MA), and other hard phases, and their role in improving the HAC resistance, have been reported [18,19]. Nevertheless, there are uncertainties regarding the relationship between the alloying elements in the steel and the HAC behaviors, primarily because of the lack of systematic efforts to understand the HAC behaviors, in terms of the hydrogen diffusion/trapping behaviors in the steel matrix and corrosion behaviors on the steel surface.

Theoretically, hydrogen atoms, formed by corrosion reactions on the steel surface, can diffuse easily into the steel matrix with a body-centered cubic (BCC) structure, and become trapped at several metallurgical defects with high binding energy [20,21,22]. According to the internal pressure and de-cohesion theories, proposed by Zapffe et al. [23], and Troiano [24], respectively, cracks were initiated by the continued accumulation of hydrogen atoms into local areas and their recombination reaction (H + H → H_2_), and propagated easily through the embrittled regions formed by a weakening of the binding force between Fe-Fe atoms. Therefore, clarification of the physical nature of hydrogen diffusion and trapping phenomena is critically important. Moreover, the other important feature of HAC, occurring in sour environments, is the corrosion behaviors on the steel surface. In particular, the amount of hydrogen infusion and its infusion kinetics can also be controlled by the characteristics of the corrosion products formed on the steel surface [25,26]. Kim et al. [26] investigated the combined addition of Cu and Ni on sour corrosion and the HIC resistance of A516 steel in sour environments. On the other hand, some alloying elements can have both beneficial and detrimental effects on sour corrosion and HAC resistance of steels in sour environments. Hence, a further in-depth study is required.

In this study, the HAC resistance of A516 steel, used in pressure vessel facilities, was investigated systematically, in terms of microstructural modifications, hydrogen diffusion/trapping behaviors, and surface characteristics, which were dependent on the addition of alloying element of C and Mo to the steel. Based on the experimental and analytical results, a desirable alloy design concept for improving the resistance to HAC is suggested.

## 2. Experimental

### 2.1. Specimen Preparation and Microstructure Observation

The test material used in this study was a low carbon steel equivalent to an ASTM A516-65 grade pressure vessel steel plate. Table 1 lists the chemical composition and mechanical properties of the three tested steel samples. The major differences in the chemical composition among the three samples are the C and Mo contents. The steel was austenitized by heating to 910 °C for 10 min and cooled to room temperature in air.

For microstructural analysis, the samples were polished to 1 μm and etched with a 5% nital solution (a mixture of 5% nitric acid and ethanol). They were then observed by field emission-scanning electron microscopy (FE-SEM) (Hitachi, Tokyo, Japan). The fractions of pearlite in the samples were measured by image analysis via optical microscopy (OM) (Zeiss, Jena, Germany). The morphological features of the precipitates in the microstructures were observed by transmission electron microscopy (TEM) (SELMI, Sumy, Ukraine) using the extraction replica method.

### 2.2. Hydrogen Induced Cracking Test (HIC)

HIC sensitivity of three samples was evaluated by the HIC experiment in reference to the National Association of Corrosion Engineers (NACE) TM 0284-96A standard [27]. Prior to conducting the experiment, the samples with 100 mm × 20 mm × 15 mm were cleaned ultrasonically in acetone. The samples were then immersed in a NACE solution (5% NaCl + 0.5% CH_3_COOH) saturated fully with H_2_S gas for 96 h. Subsequently, the level of HIC in the samples was evaluated, using an ultrasonic testing (UT) method, and the crack area ratio (CAR) was derived using Equation (1):

(1) $$\mathrm{CAR}=\frac{\sum Crack\;area}{Total\;area}\times 100\;(\%).$$

An ultrasonic inspection, a hardness test and microstructure observation were performed according to the thickness direction, in order to determine the location of cracks and microstructural properties along the thickness direction.

### 2.3. Electrochemical Hydrogen Permeation Test

The hydrogen diffusion behaviors in the steel samples were evaluated using an electrochemical permeation experiment in reference to the ISO 17081 [28] standard. The samples of the sheet-type steel membrane with a 1 mm thickness were prepared by mechanical polishing with SiC paper (P2000 grit sand paper). The polished samples were then cleaned ultrasonically in ethanol and distilled water to remove the impurities on their surfaces. It is generally considered that there is no change in the structure of the ferritic steel during sample preparation. The prepared sample was placed in the center of the permeation cell. Prior to the permeation measurement, a thin palladium (Pd) coating layer, approximately 100 nm thick, was deposited electrochemically on the sample surface in the detection side of the cell. The sample in the detection side was polarized at a constant potential of 270 mV_SCE_ in a deaerated 0.1 M NaOH solution. After reaching a background current density below 1 μm/cm^2^, a constant cathodic current density of 1 mA/cm^2^ was applied to the sample in the charging side filled with a charging solution (NACE + 0.05 M Na_2_S + 0.3 wt.% NH_4_SCN). Na_2_S was added to simulate the H_2_S atmosphere at the laboratory level because of the toxic risk of H_2_S. As the measured permeation current (rising transient) reached a steady-state value, the application of a cathodic charging was stopped and the charging solution was drained immediately for the desorption process. Information about the test procedure is reported elsewhere [29,30,31].

Preliminary tests showed that the reproducibility of the measured permeation flux in the rising transients is seldom obtained. Instead, the permeation flux in the decay transients was much more reproducible. For this reason, the apparent hydrogen diffusivity of the three samples was obtained by curve fitting with the theoretical Equation (2) [32] to the experimental permeation curves in the decay transients.

The hydrogen current decay phase,

(2) $$\frac{i_{t}-i_{0}}{i_{0}-i_{\infty }}=1-\;\frac{2L}{\sqrt{\pi Dt}}\sum_{n=0}^{\infty }exp\left[-\frac{{\left(2n+1\right)}^{2}L^{2}}{4Dt}\right]$$

where *D*, *i_t_*, *i*_0_, and *i*_∞_ are the apparent hydrogen diffusion coefficient, measured hydrogen permeation current density at time *t*, initial hydrogen permeation current density, and steady-state hydrogen permeation current density for each phase, respectively. While, *L* is the thickness of the steel membrane, and the number *n* of 0, 1, 2, 3 could be generally taken for accurate curve fitting.

Two consecutive permeation experiments, involving 1st permeation—Desorption—2nd permeation, were normally conducted to measure the irreversibly trapped hydrogen concentration in the sample [28]. On the other hand, it was difficult to completely exclude the effect of corrosion products with a low electrical conductivity [33], which can be formed on the surface during desorption, on subsequent hydrogen reduction, ad/absorption, and diffusion in the steel matrix during the second permeation. Therefore, the authors attempted to obtain the irreversibly trapped hydrogen concentration in the sample using only a first permeation curve (rising and decay transients) based on the assumption that the irreversible trapping phenomenon can contribute only to the rising transient. Therefore, the irreversibly trapped hydrogen concentrations (*C_irr_*) in the samples were estimated quantitatively by measuring the area between the two curves of the fitted decay transients with the two *D_app_* values, obtained using Equation (3), describing the rising transient and experimental decay transient.

The hydrogen permeation phase:

(3) $$\frac{i_{t}-i_{0}}{i_{\infty }-i_{0}}=\frac{2L}{\sqrt{\pi Dt}}\sum_{n=0}^{\infty }exp[-\frac{{\left(2n+1\right)}^{2}L^{2}}{4Dt}].$$

The total hydrogen trap density values (# of trap site/unit volume) of the samples were determined using Equation (4) [34],

(4) $$\frac{D_{L}}{D_{\tau }}-1=\frac{3N_{T}}{\langle C\rangle }$$

where *D_L_*, *D_τ_*, *N_T_*, and <*C*> are the lattice diffusion coefficient of hydrogen, diffusion coefficient of hydrogen in the presence of traps, the number of trap site per unit volume, and average hydrogen concentration (=J_ss_L/D_app_) obtained from rising transient, respectively. While, *D_L_* was determined to be 3.25 × 10^−9^ m^2^/s, which was obtained from American Petroleum Institute (API) grade carbon steel [35]. This value was slightly smaller than that (7.5 × 10^−9^ m^2^/s) obtained from pure iron [36]. Such differences could be caused by the fact that C atoms at the interstitial lattice sites and some substitutional atoms (C, Cr, Mo, Cu, and Ni), which may form a stress field inside the lattice, can delay hydrogen diffusion in the steel [34,37].

### 2.4. SSC Test and Fracture Surface Observation

An SSC experiment was conducted based on NACE TM 0177 [38], and tensile stresses of 85 and 95% vs. yield strength of the steel were applied using the dead-weight method. At the same time, the samples were exposed to a NACE solution fully saturated with H_2_S for 720 h. Subsequently, the SSC susceptibility was evaluated by measuring the time to rupture. Field emission scanning electron microscopy (FE-SEM) was performed to observe the fracture surfaces of the samples.

### 2.5. Corrosion Characteristic Analysis

Immersion tests were also conducte4d in the NACE solution with 0.05 M Na_2_S for four weeks to analyze the effects of corrosion and corrosion products on the surface of the samples on the SSC properties. After conducting the tests, the cross-sectional images of the samples were observed by FE-SEM. X-ray diffraction (XRD) (Bruker, Karlsruhe, Germany) was also performed to characterize the phases in the corrosion product layer formed on the steel surface.

The nature of corrosion products formed on the sample with the lowest resistance to SSC was characterized by glow discharge spectroscopy (GDS) and X-ray photoelectron spectroscopy (XPS). GDS analysis was conducted using a Leco (St. Joseph, MI, USA) GDS-850A spectrometer with Ar plasma equipped with an RF lamp. The diameter of the analysis area was 4 mm. XPS (VG Scientific Escalab 250 (Waltham, MA, USA)) was performed using monochromatic Al K-alpha radiation (1486.6 eV) with a 500 μm diameter spot size. A constant analyzer energy mode with 150 eV for the survey and high-resolution spectra was used.

## 3. Results and Discussion

### 3.1. Microstructure Observation

**Figure 1:**
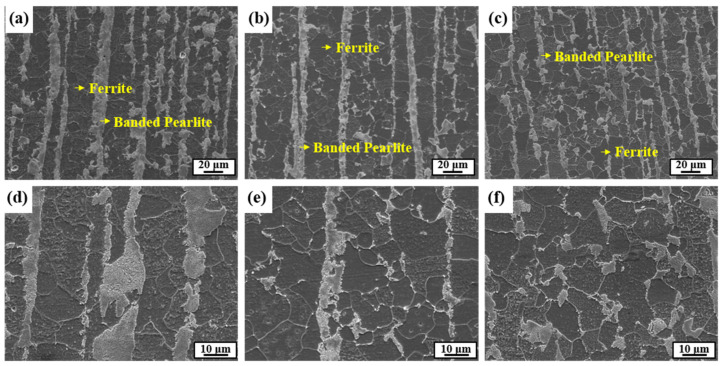
Microstructures observed by FE-SEM: (**a**,**d**) Steel A, (**b**,**e**) Steel B, and (**c**,**f**) Steel.

**Figure 2:**
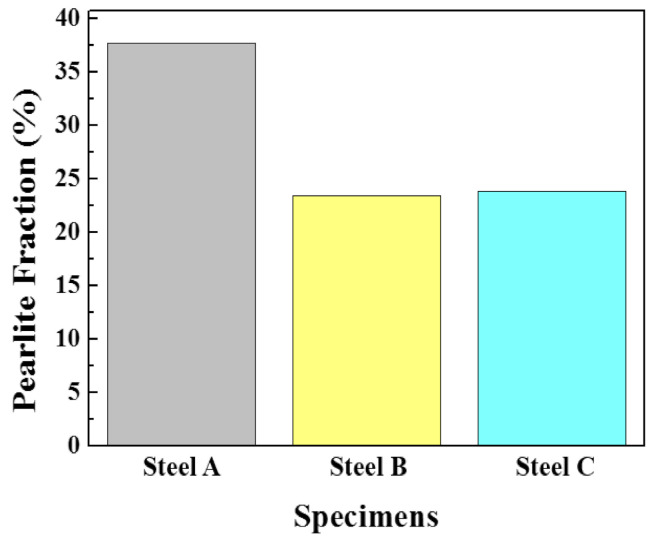
Fractions of pearlite in the microstructures of the three samples, which were measured using an image analyzer in OM.

Figure 1 presents FE-SEM images of the microstructures of the three samples. They were composed of typical ferrite and band-shaped pearlite, but the noticeable differences among the samples were the fraction, thickness, and distribution of pearlite. As expected from the C contents in the samples, the fraction of pearlite in Steel A was higher than that in the other two samples (Figure 2). There were also differences between Steel B and C with relatively low fractions of pearlite. Compared to Steel B, Steel C showed a high degree of pearlite dispersion in the microstructure, meaning that the pearlite banding index, defined previously [39,40], of Steel C was lower. A high degree of pearlite dispersion in Steel C is closely associated with the addition of Mo in the steel. According to previous studies [41], the addition of Mo in the low C steel lowers the transformation temperature of γ→α, leading to larger nucleation sites, and suppresses the diffusion of C during the cooling process due to the increased super-cooling. A more dispersed banded structure in steel with increasing Mo content was also reported [42]. The dispersion of pearlite in the microstructure has two opposite effects on hydrogen assisted cracking (HAC) of steels. The larger interface between ferrite/banded pearlite, which acts as a reversible trap for hydrogen atoms, leads to increased diffusible hydrogen contents and has a detrimental effect. The other is a beneficial effect that arises through the limited propagation path of hydrogen induced crack (HIC). In addition to these two factors, there are a variety of metallurgical parameters that affect the HAC of steels [42,43]. Further mechanistic discussion, based on the test results of HAC is needed, which will be presented in the following sections.

### 3.2. Hydrogen Induced Cracking (HIC) Test

**Figure 3:**
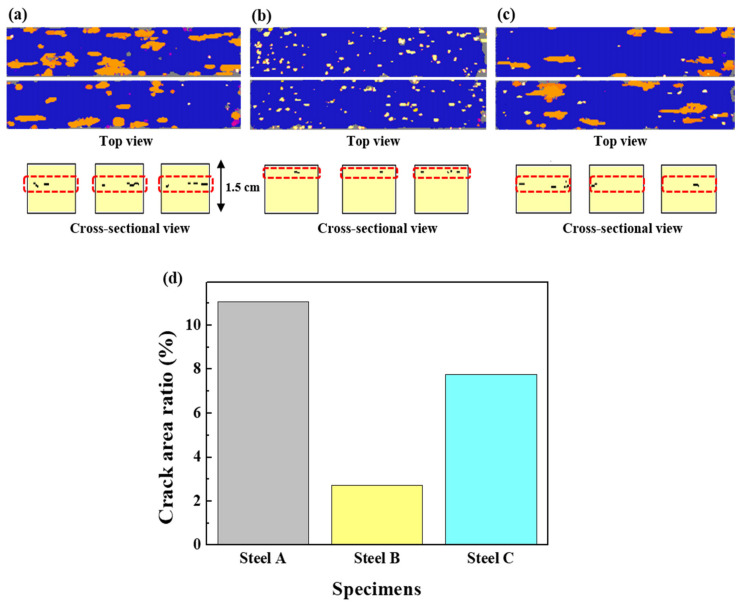
HIC test results obtained by ultrasonic detection: (**a**) Steel A, (**b**) Steel B, (**c**) Steel C, and (**d**) CAR values of the three samples.

**Figure 4:**
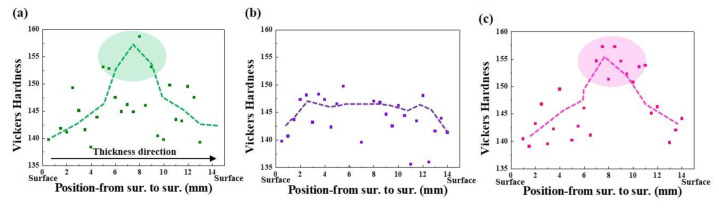
Through-thickness hardness distribution: (**a**) Steel A, (**b**) Steel B, and (**c**) Steel C.

**Figure 5:**
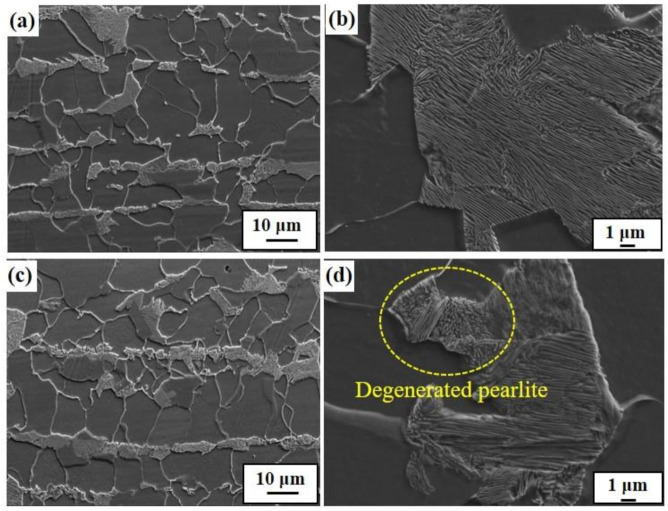
Microstructure observation of Steel C using FE-SEM: (**a**) surface region, (**b**) magnified image of (**a**), (**c**) center region, and (**d**) magnified image of (**c**).

Figure 3 presents the distribution and level of the occurrence of HIC of three samples, which were inspected ultrasonically after the HIC standard test [27]. In terms of the crack formation patterns among the samples, Steel B showed somewhat different characteristics, compared to the other two samples. A large number of fine cracks formed mostly in a quarter of Steel B in the thickness direction, which is known as the region where inclusions are mainly formed [44]. In contrast to the sample, much coarser cracks were formed mostly in the center of Steels A and C, presuming that Steels A and C had much lower resistance to the propagation of HIC. The crack sensitivity index, expressed as the CAR, increased in the order of Steel B, Steel C, and Steel A. The susceptibility of Steel A to HIC can be understood partly by the higher fraction of pearlite, with high banding properties in its microstructure, as the banded pearlite can act as a main crack propagation path. Moreover, considering that the interface between ferrite/cementite can act as a reversible trap for hydrogen (11–17 kJ/mol [45,46]), a higher fraction of pearlite in Steel A can provide a larger interfacial area for hydrogen trapping, facilitating embrittlement and cracking. Contrary to expectations, Steel C, with a high degree of pearlite dispersion, was more susceptible to HIC than Steel B. This suggests that the other factors may have been involved in the occurrence of HIC. It is notable that the locations of crack formation in the two samples were different. Similar to Steel A, Steel C had coarse cracks formed at the center region in the thickness direction. From a metallurgical point of view, the microstructure of the center region of the steel plates is somewhat different from that of surface region owing to the centerline segregation phenomenon, and some low-temperature transformation phases with high hardness can be more segregated in the center region of the steel plates. For a more precise understanding, the Vickers hardness of the three samples was measured in the thickness direction, and the morphological features of pearlite formed in surface and center regions of the samples were observed (Figure 4). In the case of Steel A and C, the mid-thickness regions had higher hardness than the top and bottom regions. On the other hand, no high hardness values were particularly measured in the center regions of Steel B. Considering the inverse relationship between the hardness level and HIC resistance [47], the cause of the higher CAR and much more prominent crack propagation in the mid-thickness region of Steel C, compared to Steel B, is better understood. The detrimental effects by local areas with high hardness in the mid-thickness region overtake the beneficial effect by a high degree of pearlite dispersion in the microstructure of Steel C, resulting in lower resistance to HIC. The presence of local areas with high hardness in the mid-thickness region of Steel A or C can also be understood by the degenerated pearlite formed in the mid-thickness region of Steel A or C, as shown in Figure 5. The degenerated pearlite, which is generally referred to as pseudopearlite [48], can be formed under the condition of limited C diffusion during the cooling process. Lee et al. [49] reported that the transformation temperature of the 2nd phase (pearlite) decreased sharply from approximately 600 °C to 500 °C by the addition of Mo in low alloy steel. Hence, C diffusion was not quick enough to bring about the complete growth of pearlite. Based on these facts, it is expected that an increase in C and Mo in these low C steels can form the degenerated pearlite with a higher hardness in the mid-thickness region due the center-segregation, thereby resulting in a lower HIC propagation resistance. These HIC properties are also dependent on hydrogen diffusion and trapping behaviors in steels. These behaviors are discussed in more detail in the following section.

### 3.3. Electrochemical Hydrogen Permeation Test

**Figure 6:**
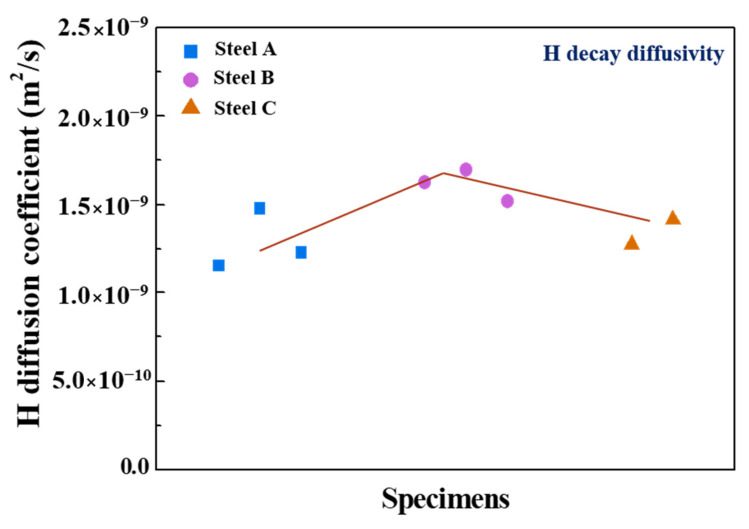
Hydrogen diffusion coefficients obtained by curve fitting to decay permeation curves.

**Figure 7:**
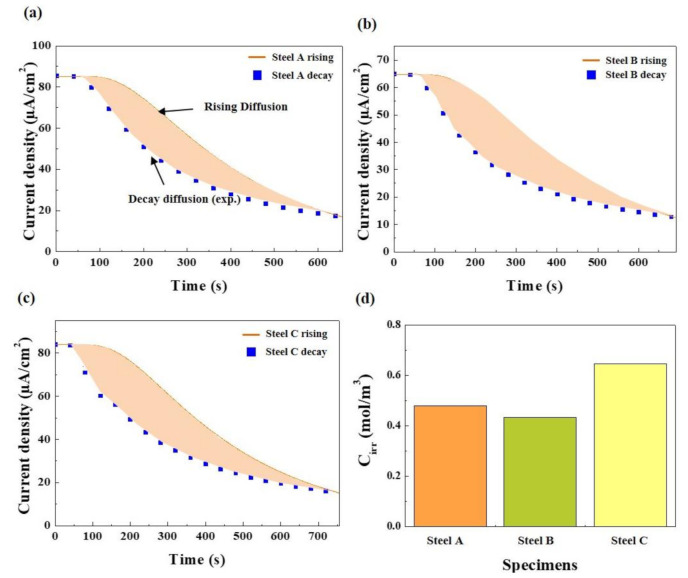
Irreversibly trapped hydrogen contents measured by the area between the two fitted decay transients: (**a**) Steel A, (**b**) Steel B, and (**c**) Steel C, and (**d**) bar chart of the irreversibly trapped hydrogen contents of the three steel samples.

**Figure 8:**
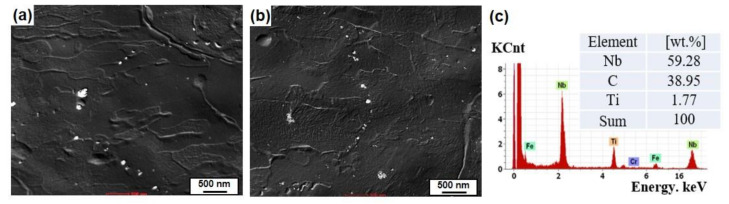
TEM images showing fine-sized precipitates in the steel matrix: (**a**) Steel B and (**b**) Steel C, and (**c**) EDS analysis of fine-sized precipitates.

Figure 6 presents the hydrogen diffusion coefficients of three samples, obtained by curve fitting to the experimental decay transients. The diffusion coefficients increased in the order of Steel A, Steel C, and Steel B. As mentioned above, the more extensive interface between ferrite/pearlite acting as a reversible trap [45,46] of Steel A can decrease the diffusion kinetics of hydrogen in the steel. The highest diffusion coefficient of Steel B can be understood in this context. On the other hand, the diffusion coefficient of Steel C with a similar pearlite fraction to that of Steel B was lower than expected. Here, the mechanistic reasons for this phenomenon are proposed as follows. A comparatively lower diffusivity in Steel C may be closely associated with the distribution of the banded structure. More dispersed banded pearlite can provide a larger interfacial area for hydrogen trapping, leading to slower hydrogen diffusion kinetics. The formation of a stress field in the lattice structure of Steel C could also be one of the plausible reasons. Hydrogen can diffuse into areas where the stress field is set up [34,37]. The stress field in the lattice structure, which was formed by the difference in atomic size between Fe and Mo, can also delay hydrogen diffusion/transport with the hydrogen binding energy of 26–27 kJ/mol [37,45]. These factors are somewhat speculative, and their impacts on the hydrogen diffusion kinetics may be insignificant. The other factors may also be involved in the diffusion kinetics. In this context, the irreversible trapping phenomenon was also evaluated, as shown in Figure 7. The irreversibly trapped hydrogen contents (*C_irr_*) in Steel A and B showed similar levels within the error range, whereas a much higher level of *C_irr_* was obtained in Steel C with a higher Mo content. This suggests that additional traps associated with Mo could act as an irreversible trap for hydrogen, leading to slower hydrogen diffusion kinetics. For a more precise understanding, TEM analysis was conducted on Steel B and C, and the results are shown in Figure 8. The fine-sized precipitates of (Ti, Nb) C, classified as an irreversible trap for hydrogen [50], were identified in both steels. Although, it appears that the precipitates observed in Steel C were slightly smaller than those observed in Steel B, their density and distribution cannot be discussed from these limited data. On the other hand, no Mo-based precipitate was observed. Considering that it is generally difficult for fine precipitates, several nanometers in size, to be extracted using the replica method, Mo-based precipitates are too fine to be detected easily. In this case, it would be better for the TEM observation to be conducted with the thin film sample. Although, the nature of the irreversible traps was not clearly characterized in this study, a few comments are noteworthy. Steel C had the largest amount of irreversibly trapped hydrogen among the steels, which was closely associated with the presence of fine precipitates, resulting in slower diffusion kinetics of hydrogen. 

**Figure 9:**
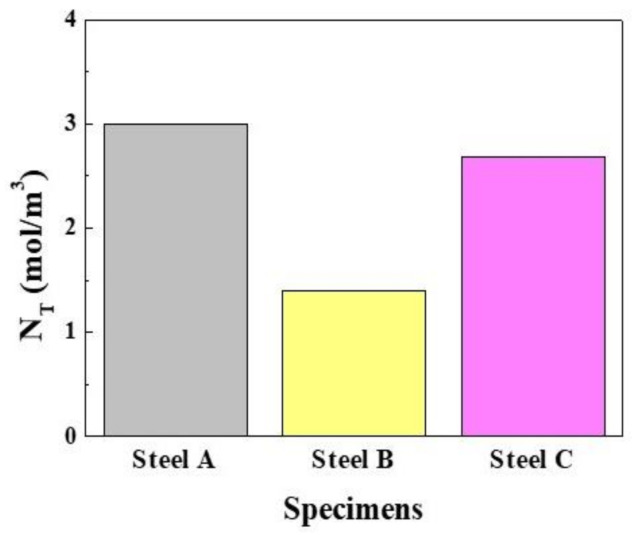
Total hydrogen trap density in the steel.

Figure 9 shows the overall hydrogen trap density (*N_T_*), considering both reversible and irreversible trapping, which was derived from Equation (4). The highest *N_T_* of Steel A can be understood by the higher fraction of pearlite, which corresponds to the lowest diffusivity of hydrogen in Steel A, suggesting that the C content in steels can be a crucial element increasing the hydrogen trap density and reducing the hydrogen diffusivity. On the other hand, the *N_T_* of Steel C was between those of Steel A and B. The *N_T_* of Steel C, which was higher than expected, was attributed to be due to the high level of irreversibly trapped hydrogen in the steel, as discussed above. In general, the presence of a higher density of irreversible traps in steel is considered to be due to a higher resistance to HIC as the traps with a high hydrogen binding energy immobilize the diffusible hydrogen and suppress the local hydrogen concentrations around potential crack areas [51]. As shown above, however, the HIC resistance of Steel C was much lower than that of Steel B. In relation to the effects of irreversible traps on the resistance to HAC in steels, there are additional factors that need to be considered, such as size [52], shape [53], and coherency with the matrix [54]. These also suggest that the HAC resistance cannot be evaluated simply by the hydrogen diffusion/trapping parameters obtained from the electrochemical permeation experiment. Hence, further in-depth analysis is required.

### 3.4. Sulfide Stress Corrosion Cracking (SSC) Test

**Figure 10:**
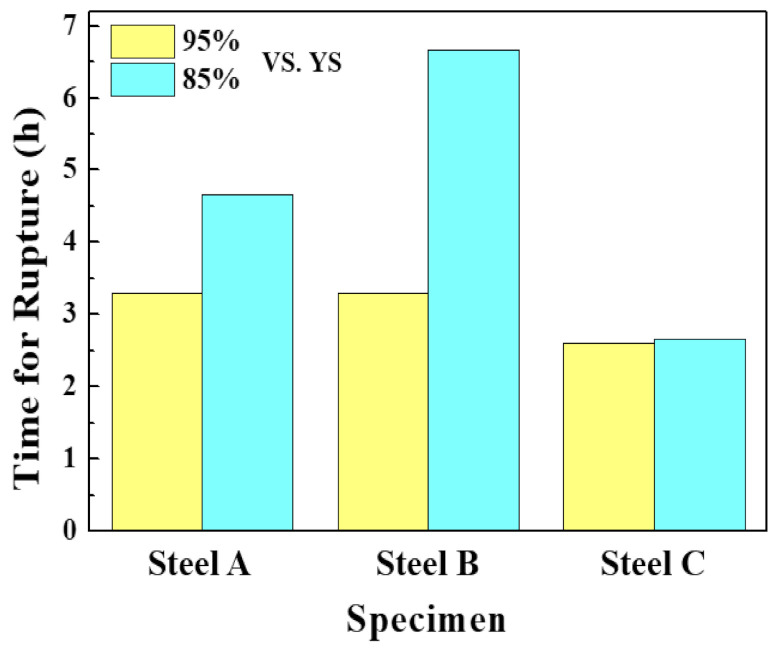
Time for rupture of the three samples, obtained by the SSC test.

The SSC resistance was also evaluated, and Figure 10 presents the time to rupture of the samples after the SSC test which was conducted in reference to NACE TM0177 [38]. In the three samples, the time to rupture decreased with increasing applied stress. Under applied stress of 95% vs. YS of steel, there was no significant difference in the time to rupture among the samples. On the other hand, the time to rupture under an applied stress of 85% versus YS of steel decreased in the order of Steel B, Steel A, and Steel C. The highest SSC resistance of Steel B is understandable based on the HIC resistance, Vickers hardness distribution, and hydrogen diffusivity/trap density, as shown above.

**Figure 11:**
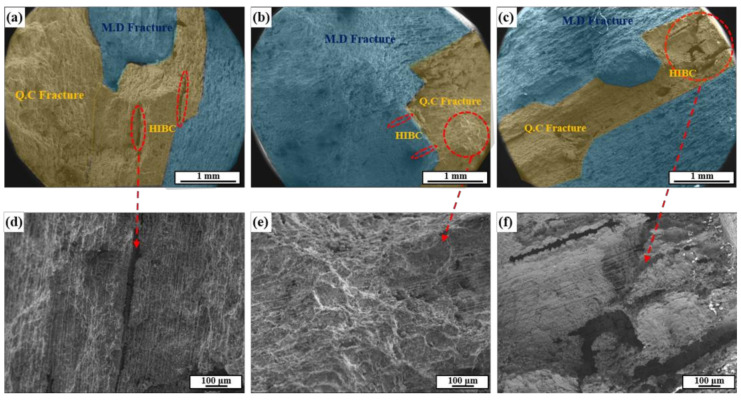
Fracture surface morphologies of the three samples after the SSC test: (**a**) Steel A, (**b**) Steel B, and (**c**) Steel C, and (**d**–**f**) magnified images of the circle marked on (**a**–**c**), respectively.

Regarding the fracture surface showing the micro-dimple (MD) and quasi-cleavage (QC) patterns (Figure 11), in Steel B, the MD/QC ratio was the largest and the size of the hydrogen induced blister crack (HIBC) in the fracture surface was the smallest. On the other hand, the time to rupture of Steel C was much shorter than expected. From the fracture surface observations, the MD/QC ratio of Steel C was larger than that of Steel A, suggesting that the embrittlement index of Steel C was slightly lower than that of Steel A. On the other hand, it is notable that surface degradation as a form of local-pit like corrosion was observed in Steel C. The major difference between the HIC and SSC experiments lies in the presence of applied stress. Considering the relationship between the applied stress and corrosion behaviors of steels in sour environments [55], the reason for the lowest SSC resistance of Steel C could be closely related to the surface properties. Based on the observations of the fracture surface of Steel C, it appears that pit-like corrosion on the surface acting as a stress intensifier under the applied stress conditions was connected to the internal HIBC, facilitating rupture. Therefore, it is surface defects, such as local-pit like corrosion, can make Steel C more susceptible to SSC, and the corrosion behaviors of Steel C containing Mo should be analyzed further.

### 3.5. Corrosion Product Analysis

**Figure 12:**
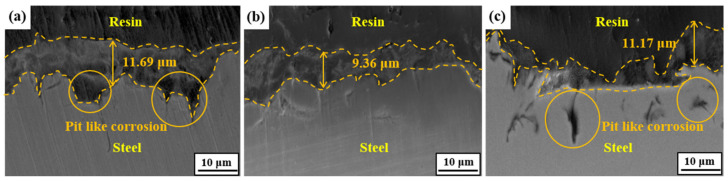
Cross-sectional images after immersion in a NACE solution with 0.05 M Na_2_S for four weeks: (**a**) Steel A, (**b**) Steel B, and (**c**) Steel C.

Figure 12 showed the cross-sectional images of the samples after immersing in a NACE solution containing 0.05 M Na_2_S for four weeks. The corrosion product formed on Steel A was thicker than Steel B and C. The highest growth rate of corrosion products formed on Steel A can be attributed to the highest rate of anodic dissolution, considering the positive relationship between the rate of precipitation and the amount of Fe^2+^ supplied by the anodic dissolution of steel [56]. From the viewpoint of electrochemical corrosion, the Fe_3_C contained in steels acts as a cathode, and a higher fraction and larger size of the cathode lead to a higher dissolution rate of the anode (Fe matrix) [57]. On the other hand, it appears from the cross-sectional observation of Steel C that the corrosion products were formed unevenly, presuming that some portions of products were detached locally, and localized pitting occurred below the corrosion products. Preliminary tests showed that the corrosion product formed on Steel C had poor adhesion to the steel substrate, and the weight loss rate measured after three weeks of immersion was the highest in Steel C.

**Figure 13:**
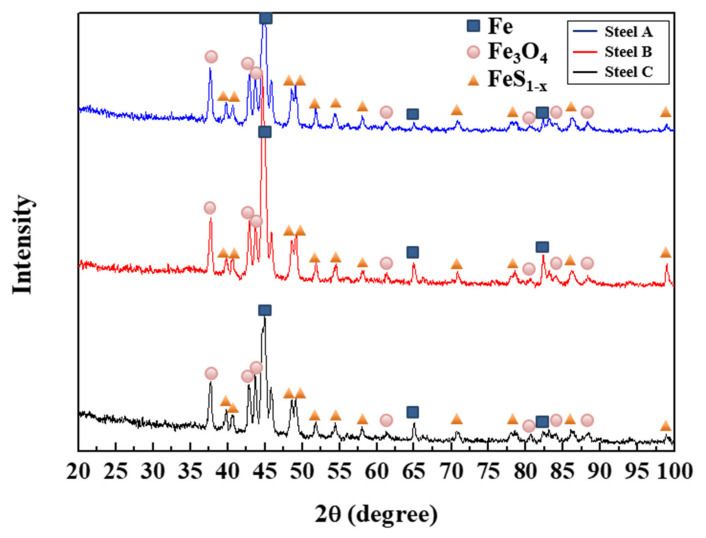
XRD analysis on the corrosion product layer formed on the steel surface after immersion test in a NACE solution with 0.05 M Na_2_S for four weeks.

**Figure 14:**
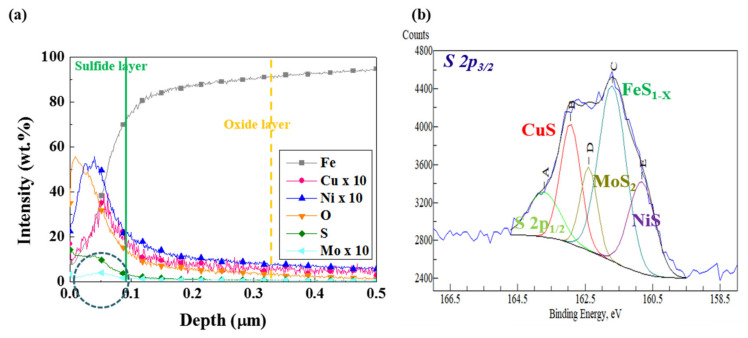
(**a**) GDS analysis, and (**b**) XPS analysis of the corrosion product layer formed on Steel C after the immersion test in a NACE solution with 0.05 M Na_2_S for four weeks.

Although XRD analysis (Figure 13) showed that the corrosion products that formed on all three samples consisted mainly of Fe_3_O_4_ and FeS_1−x_, there is some doubt as to whether the Mo in Steel C can adversely affect the stability of the corrosion products on the surface. Therefore, additional analyses of the corrosion products formed on Steel C were conducted. GDS and XPS analyses (Figure 14) showed that the corrosion products were composed of a mixture of oxides and sulfides. Based on XPS analyses, the binding energies and relative quantity of compounds for S 2p3/2 spectra of the corrosion product formed on Steel C were obtained, and they are listed in Table 2. The sulfides were composed mainly of FeS_1−x_, CuS, NiS, and MoS_2_. A previous study [26] reported that the small addition of Cu and Ni to steels leads to the formation of thin and dense corrosion products in sour environments, and contributes to the enhanced corrosion resistance. Accordingly, local detachment of corrosion products with uneven interfaces, and localized pitting of Steel C may have resulted from the formation of MoS_2_. Koh et al. [58] pointed out that the formation of MoS_2_ in iron sulfide based corrosion products formed on the low C steel exposed to a sour environment weakens the stability of the sulfide products, due to the differences in their crystal structures and lattice parameters. Based on these facts, the desirable alloy design strategy to enhance the HAC of A516-65 steel is proposed as follows. It is preferable that the C content should be reduced, in order to decrease the fraction of pearlite. Moreover, the optimal Mo content providing only a beneficial effect (i.e., dispersion of banded structure) on the HAC needs to be determined.

## 4. Conclusions

The effects of alloying elements (C and Mo) on the HAC properties of ASTM A516-65 grade steel use, as pressure vessel facilities, were investigated using a range of experimental and analytical methods. The main conclusions are as follows:

1. The microstructures of the three steel samples (Steel A, B and C) were composed of ferrite and band-shaped pearlite. A higher C content in the steel resulted in a higher fraction of banded pearlite. On the other hand, the addition of Mo contributed to the dispersion of the banded structure.
2. The sample with lower C and Mo contents (Steel B) showed the highest resistance to HIC, with fine cracks initiated mostly in a quarter of the sample in the thickness direction. In contrast, much coarser cracks were formed mostly in the center of the other samples with higher C, and Mo contents (Steel A and C), respectively, and they showed higher susceptibility to HIC. This is closely associated with the difference in hardness distribution in the through- thickness direction caused by the center segregation phenomenon.
3. The sample with lower C and Mo contents and a smaller fraction of pearlite (Steel B) had the highest diffusion coefficient of hydrogen. On the other hand, the diffusion coefficient of the sample with a higher Mo content (Steel C) was rather high and similar to the case of the sample with a higher C content (Steel A). Therefore, more dispersed banded pearlite, which provides a larger interfacial area for hydrogen trapping, the formation of a stress field in the lattice structure by the difference in atomic size between Fe and Mo, and the presence of irreversible trap site for hydrogen, are the proposed mechanistic reasons.
4. The sample with a higher Mo content (Steel C), however, showed the shortest rupture time by the SSC experiment. In contrast to the fracture surfaces of the other samples (Steel A and B), pit-like corrosion occurred on the surface of Steel C. This was connected to the internal HIBC, which facilitates rupture under the applied stress conditions. The corrosion products formed on Steel C, which were composed of FeS_1−x_, MoS_2_, CuS, and NiS have an uneven interface with the steel substrate and were locally detached. These results suggest that the addition of Mo in the steel should be optimized further to improve the resistance to HAC in sour environments.

## Figures and Tables

**Table 1:** Chemical compositions and mechanical properties of the three tested steel samples.

Specimens	Chemical Composition (wt.%)								YS (MPa)	TS (MPa)	Strain (%)
	C	Mn	Si	Mo	Cr	Nb	Ti	Fe			
Steel A	0.16	1–1.5	0.3–0.4	<0.003	<0.05	<0.01	<0.01	Bal.	349.11	512.18	16.22
Steel B	0.13	1–1.5	0.3–0.4	0.01–0.015	<0.05	<0.01	<0.01	Bal.	345.1	487.21	17.55
Steel C	0.13	1–1.5	0.3–0.4	0.05–0.055	<0.05	<0.01	<0.01	Bal.	347.33	492.61	17.22

**Table 2:** Binding energies and relative quantity of compounds for S 2p_3/2_ spectra of Steel C after immersion for four weeks.

Phase	Binding Energy (eV)	Relative Quantity
S 2p_1/2_	163.7	0.095 (±0.009)
CuS	162.8	0.207 (±0.021)
MoS_2_	162.3	0.100 (±0.009)
FeS_1-X_	161.66	0.374 (±0.037)
NiS	160.79	0.224 (±0.022)
